# Supplementary material for: Entamoeba histolytica: In Silico and In Vitro Oligomerization of EhHSTF5 Enhances Its Binding to the HSE of the EhPgp5 Gene Promoter
Source: Int J Mol Sci. 2024 Apr 11;25(8):4218. doi: 10.3390/ijms25084218 (PMC11050682; doi:10.3390/ijms25084218)
Supplement: Supplementary file 1 [file ijms-25-04218-s001.zip › Figures (S1 to S3) and Supplementary Table S1.pdf]

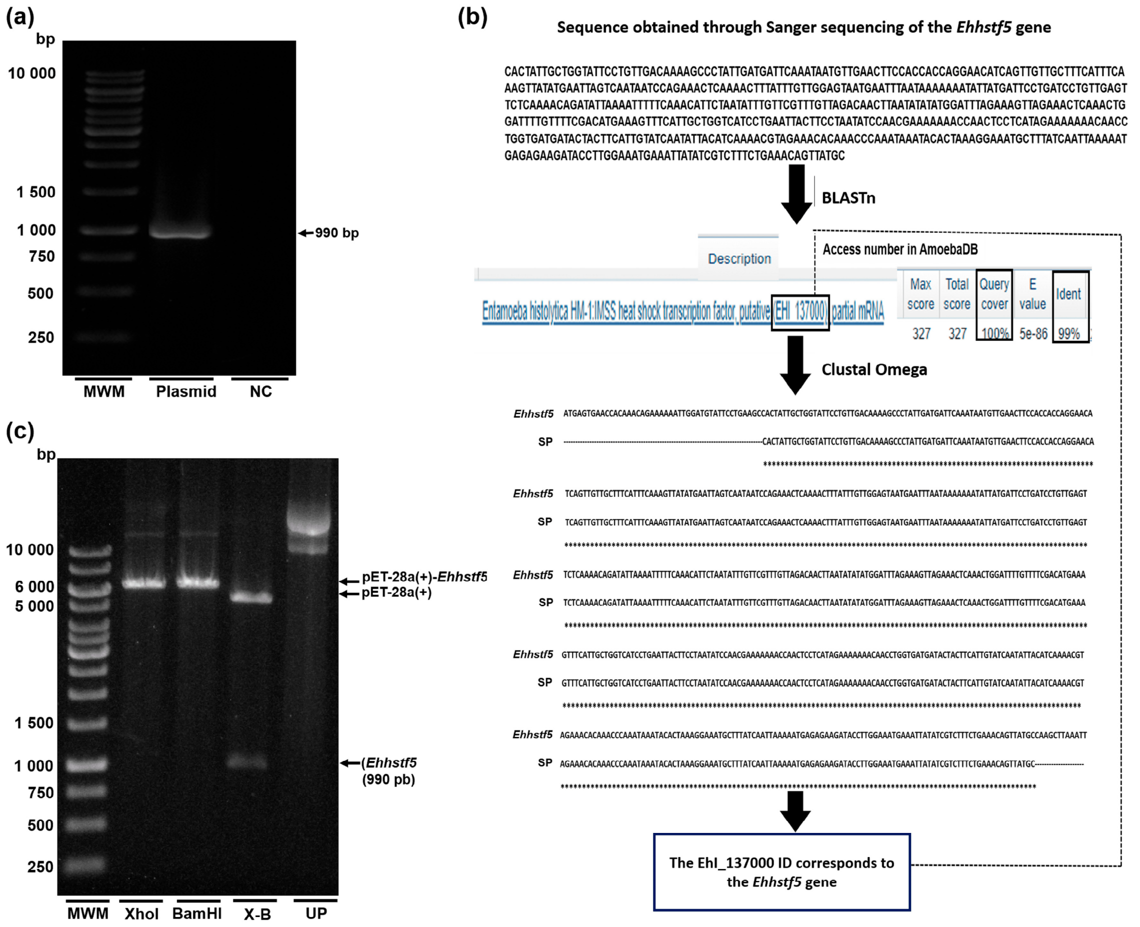


**Supplementary Figure S1.** The insertion of the *Ehhstf5* gene into the pET-28a(+) plasmid was confirmed by PCR, obtaining a product of approximately 990 base pairs (bp) (a). This product was subsequently sequenced using Sanger sequencing. The sequenced product (SP) was analyzed using BLASTn, showing alignment with the *Ehhstf5* gene (ID EHI_137000). Clustal Omega analysis comparing the sequenced product with the AmoebaDB-derived sequence showed consistent results, with 99% identity and 100% homology. (b). Enzymatic restriction assays were additionally conducted using the plasmid. When XhoI or BamHI enzymes were applied separately, the plasmid linearized, revealing an approximate size of 6000 bp. Simultaneous application of both enzymes (X-B) resulted in the release of the Ehhstf5 gene, with a size close to 1000 bp, and the plasmid reduced to approximately 5000 bp. As a negative control, the unrestricted plasmid (UP) was used, showing a size greater than 6000 bp (c). MWP; molecular weight marker, NC; negative control. In the alignment with the Clustal Omega server, asterisks (*) indicate amino acids that are identical across all sequences.

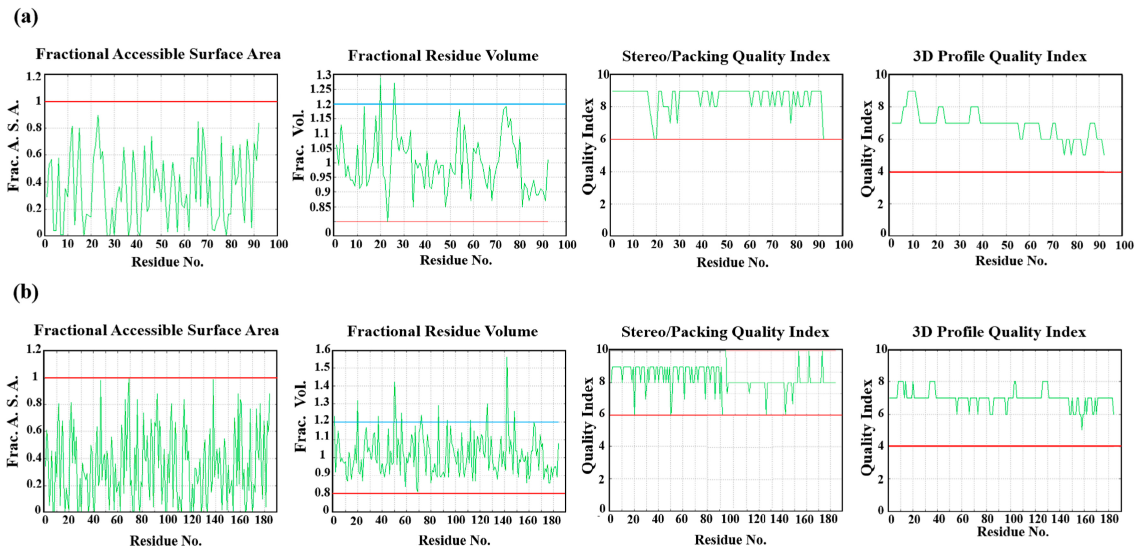


**Supplementary Figure S2**. Prediction of physicochemical properties and geometric characteristics of mEhDBD5 and dEhDBD5 Models: The VADAR server criteria indicate desirable scores below 1 for Fractional Accessible Surface Area analysis, between 0.8 and 1.2 for Fractional Residue Volume, and above 6 for the Stereo/Packing Quality Index, as well as exceeding 4 for the 3D Profile Quality Index. Notably, both mEhDBD5 (a) and dEhDBD5 (b) models achieved these target scores in all categories, underscoring their structural integrity and quality.

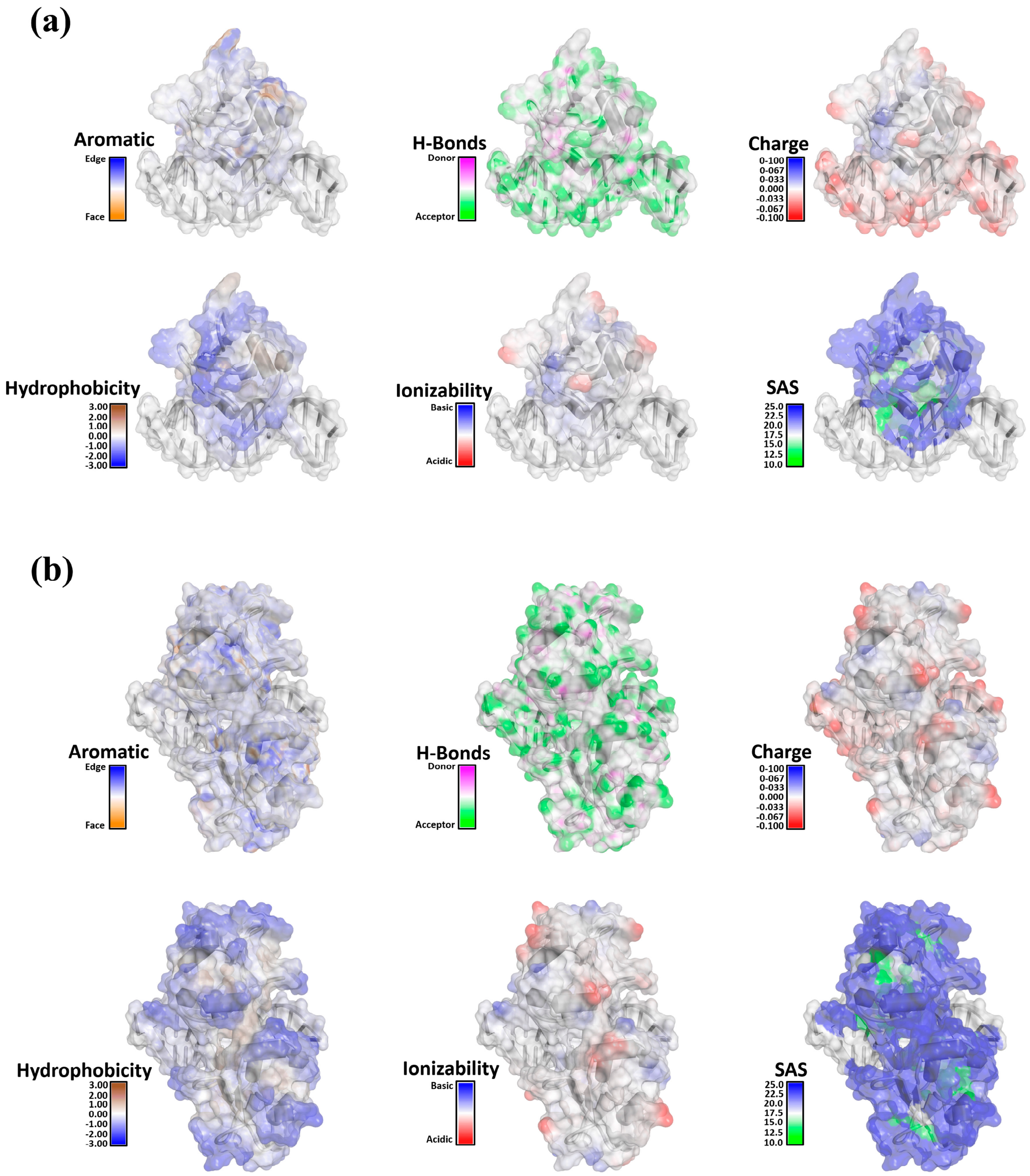


**Supplementary Figure S3**. The physicochemical properties of the dEhDBD5-HSE_*EhPgp5* complexes, both in the monomeric (a) and dimeric (b) conformations, were comprehensively analyzed using BIOVIA Discovery Studio. This analysis encompassed a range of characteristics including intra- and intermolecular aromatic interactions, the potential for hydrogen bonding, the distribution of molecular charges, degrees of

hydrophobicity, ionizability, and the solvent-accessible surface area (SAS). These properties are essential for understanding the stability of the complexes, reactivity, and potential biological interactions.

**Supplementary Table S1.** Intermolecular interaction of the mEhDBD5-HSE_*EhPgp5* and dEhDBD5-HSE_*EhPgp5* complexes

**mEhDBD5_*EhPgp5* (string 5'-3')**

**Hydrogen Bonds**

| Index | Residue | AA | Distance H-A | Distance D-A | Donor Angle | Protein donor? | Side chain | Donor Atom | Acceptor Atom |
|---|---|---|---|---|---|---|---|---|---|
| 1 | 55 (1) | ARG | 2.88 | 3.67 | 137.95 | Yes | Yes | 459 [Ng+] | 890 [Npl] |
| 2 | 55 (1) | ARG | 3.41 | 3.91 | 113.72 | No | Yes | 890 [Npl] | 457 [Ng+] |
| 3 | 55 (1) | ARG | 2.72 | 3.33 | 120.61 | No | Yes | 911 [Npl] | 457 [Ng+] |
| 4 | 58 (1) | ASN | 3.09 | 3.75 | 126.85 | No | Yes | 894 [O3] | 484 [O2] |
| 5 | 58 (1) | ASN | 2.78 | 3.7 | 156.54 | Yes | Yes | 485 [Nam] | 881 [O3] |

**Salt Bridges**

| Index | Residue | AA | Distance | Protein positive? | Ligand Group | Ligand Atoms |
|---|---|---|---|---|---|---|
| 1 | 92 (1) | LYS | 4.13 | yes | Phosphate | 896, 896, 881, 898, 915, 916 |

**mEhDBD5_*EhPgp5* (string 3'-5')**

**Hydrogen Bonds**

| Index | Residue | AA | Distance H-A | Distance D-A | Donor Angle | Protein donor? | Side chain | Donor Atom | Acceptor Atom |
|---|---|---|---|---|---|---|---|---|---|
| 1 | 1 (1) | ILE | 3.6 | 4.1 | 112.66 | Yes | No | 1 [N3] | 1202 [O3] |
| 2 | 55 (1) | ARG | 3.11 | 3.9 | 137.32 | Yes | Yes | 460 [Ng+] | 1279 [Nar] |
| 3 | 55 (1) | ARG | 3.41 | 3.96 | 117.4 | No | Yes | 1278 [Npl] | 457 [Ng+] |
| 4 | 56 (1) | GLN | 2.39 | 3.33 | 158.59 | Yes | Yes | 469 [Nam] | 1257 [Npl] |
| 5 | 60 (1) | TYR | 2.09 | 2.55 | 109.25 | Yes | Yes | 505 [O3] | 1220 [O2] |
| 6 | 91 (1) | ARG | 1.85 | 2.79 | 158.78 | Yes | Yes | 760 [Ng+] | 1236 [Npl] |
| 7 | 91 (1) | ARG | 2.3 | 3.11 | 139.27 | Yes | Yes | 761 [Ng+] | 1236 [Npl] |

**Salt Bridges**

| Index | Residue | AA | Distance | Protein positive? | Ligand Group | Ligand Atoms |
|---|---|---|---|---|---|---|
| 1 | 47 (1) | HIS | 4.21 | Yes | Phosphate | 1242, 1242, 1227, 1244, 1261, 1262 |

**dEhDBD5_*EhPgp5* (string 5'-3')**

**Hydrogen Bonds**

| Index | Residue | AA | Distance H-A | Distance D-A | Donor Angle | Protein donor? | Side chain | Donor Atom | Acceptor Atom |
|---|---|---|---|---|---|---|---|---|---|
| 1 | 48 (1) | SER | 3.48 | 4.04 | 118.53 | Yes | No | 1170 [Nam] | 1666 [O3] |
| 2 | 49D (1) | ASN | 2.49 | 3.37 | 149.61 | Yes | Yes | 1183 [Nam] | 1649 [O3] |
| 3 | 49D (1) | ASN | 3.15 | 3.73 | 119.28 | Yes | No | 1176 [Nam] | 1666 [O3] |
| 4 | 52D (1) | SER | 2.1 | 2.69 | 117.71 | Yes | Yes | 1203 [O3] | 1666 [O3] |
| 5 | 55 (2) | ARG | 3.1 | 3.44 | 101.78 | Yes | Yes | 460 [Ng+] | 1743 [O2] |
| 6 | 56D (1) | GLN | 2.14 | 2.6 | 106.83 | Yes | Yes | 1241 [Nam] | 1646 [O3] |
| 7 | 58B (2) | ASN | 2.05 | 2.95 | 150.48 | Yes | Yes | 485 [Nam] | 1708 [O3] |
| 8 | 64 (2) | LYS | 1.42 | 2.38 | 163.28 | Yes | No | 532 [Nam] | 1707 [O2] |

**Salt Bridges**

| Index | Residue | AA | Distance | Protein positive? | Ligand Group | Ligand Atoms |
|---|---|---|---|---|---|---|
| 1 | 47 (1) | HIS | 3.79 | Yes | Phosphate | 1647, 1647, 1666, 1632, 1649 |
| 2 | 63 (2) | ARG | 5.03 | Yes | Phosphate | 1709, 1709, 1728, 1711, 1695, 1727 |
| 3 | 64 (2) | LYS | 3.29 | Yes | Phosphate | 1668, 1668, 1653, 1670, 1687, 1688 |
| 4 | 92 (2) | LYS | 3.83 | Yes | Phosphate | 1709, 1709, 1728, 1711, 1695, 1727 |

**dEhDBD5_*EhPgp5* (string 3'-5')**

**Hydrogen Bonds**

| Index | Residue | AA | Distance H-A | Distance D-A | Donor Angle | Protein donor? | Side chain | Donor Atom | Acceptor Atom |
|---|---|---|---|---|---|---|---|---|---|
| 1 | 52 (2) | SER | 2.7 | 3.41 | 130.78 | Yes | Yes | 431 [O3] | 1970 [O3] |
| 2 | 52 (2) | SER | 2.68 | 3.41 | 132.26 | No | Yes | 1970 [O3] | 431 [O3] |
| 3 | 55 (2) | ARG | 3.23 | 3.89 | 125.7 | No | Yes | 1987 [Npl] | 457 [Ng+] |
| 4 | 55 (1) | ARG | 1.84 | 2.51 | 121.89 | Yes | Yes | 1232 [Ng+] | 2051 [Nar] |
| 5 | 55 (1) | ARG | 2.5 | 3.41 | 152.63 | No | Yes | 2050 [Npl] | 1229 [Ng+] |
| 6 | 56 (2) | GLN | 2.01 | 2.55 | 111.79 | Yes | Yes | 469 [Nam] | 1950 [O2] |
| 7 | 58 (1) | ASN | 2.52 | 3.5 | 171.33 | Yes | Yes | 1257 [Nam] | 2013 [O2] |
| 8 | 64 (1) | LYS | 2.27 | 3.11 | 143.41 | Yes | No | 1304 [Nam] | 2012 [O3] |
| 9 | 66 (2) | GLU | 2.99 | 3.39 | 106.89 | Yes | Yes | 557 [O3] | 2133 [O3] |

**Salt Bridges**

| Index | Residue | AA | Distance | Protein positive? | Ligand Group | Ligand Atoms |
|---|---|---|---|---|---|---|

| | | | | | | |
|---|---|---|---|---|---|---|
| **1** | 46 (1) | LYS | 5.17 | Yes | Phosphate | 2176, 2176, 2195, 2178, 2161, 2194 |
| **2** | 47 (2) | HIS | 4.84 | Yes | Phosphate | 1951, 1951, 1971, 1935, 1953, 1970 |
| **3** | 63 (1) | ARG | 4.8 | Yes | Phosphate | 2014, 2014, 1999, 2016, 2033, 2034 |
| **4** | 64 (1) | LYS | 4.85 | Yes | Phosphate | 1972, 1972, 1957, 1974, 1991, 1992 |
| **5** | 91 (2) | ARG | 4.21 | Yes | Phosphate | 1909, 1909, 1911, 1894, 1927, 1928 |
| **6** | 92 (1) | LYS | 4.92 | Yes | Phosphate | 2014, 2014, 1999, 2016, 2033, 2034 |

**The amino acids marked with numbers (1) or (2) correspond to those involved in the interaction with HSE_*EhPgp5* of monomer 1 or 2, respectively.**
